# Bacterial Approaches for Assembling Iron-Sulfur Proteins

**DOI:** 10.1128/mBio.02425-21

**Published:** 2021-11-16

**Authors:** Karla Esquilin-Lebron, Sarah Dubrac, Frédéric Barras, Jeffrey M. Boyd

**Affiliations:** a Department of Biochemistry and Microbiology, Rutgers, the State University of New Jersey, New Brunswick, New Jersey, USA; b Institut Pasteur, Université de Paris, CNRS UMR 2001, Department of Microbiology, SAMe Unit, Paris, France; Ohio State University

**Keywords:** bacteria, iron-sulfur cluster, iron, sulfide, SUF, ISC, NIF, genetic regulation, iron regulation, iron utilization, metalloproteins, metalloregulation, sulfur

## Abstract

Building iron-sulfur (Fe-S) clusters and assembling Fe-S proteins are essential actions for life on Earth. The three processes that sustain life, photosynthesis, nitrogen fixation, and respiration, require Fe-S proteins. Genes coding for Fe-S proteins can be found in nearly every sequenced genome. Fe-S proteins have a wide variety of functions, and therefore, defective assembly of Fe-S proteins results in cell death or global metabolic defects. Compared to alternative essential cellular processes, there is less known about Fe-S cluster synthesis and Fe-S protein maturation. Moreover, new factors involved in Fe-S protein assembly continue to be discovered. These facts highlight the growing need to develop a deeper biological understanding of Fe-S cluster synthesis, holo-protein maturation, and Fe-S cluster repair. Here, we outline bacterial strategies used to assemble Fe-S proteins and the genetic regulation of these processes. We focus on recent and relevant findings and discuss future directions, including the proposal of using Fe-S protein assembly as an antipathogen target.

## INTRODUCTION

Iron (Fe) is an essential nutrient for nearly all organisms. The importance of Fe for the survival of microbes is highlighted by the fact that many organisms encode multiple Fe acquisition systems. These acquisition systems aid in competition and allow cells to acquire Fe under a variety of conditions to meet demand.

A large proportion of internalized Fe is housed within inorganic prosthetic groups called iron-sulfur (Fe-S) clusters, which are utilized by organisms in the three primary branches of life. Protein Fe-S clusters are typically ligated using cysteine thiolates and are commonly found as rhombic [2Fe-2S] or cubic [4Fe-4S] clusters; however, more complex Fe-S cofactors are utilized for specialized processes such as nitrogen fixation and hydrogen metabolism. When Escherichia coli is cultured in a defined medium with glucose or acetate as a carbon source, approximately 30% of the intracellular Fe is located in Fe-S clusters and low-spin ferrous heme centers (1).

As a result of their structural and electronic plasticity, Fe-S clusters are utilized for a variety of cellular functions. The genome of E. coli is predicted to encode ∼140 Fe-S proteins (out of the ∼4,300 total protein-coding open reading frames [ORFs] [2]) that have wide-ranging functions, including carbon transformations, environmental sensing, DNA repair, and respiration (3). Likewise, the metabolisms of most organisms are highly reliant on the functionalities of the Fe-S proteins. Failure to properly maturate Fe-S proteins results in widespread metabolic disorders and in some cases can lead to cell death (4–6). Bacillus subtilis strains (lacking Suf) and E. coli strains (lacking Suf and Isc) cannot build Fe-S clusters and are nonviable because they cannot properly maturate the essential Fe-S proteins IspG and IspH, which are required for isoprenoid synthesis (7, 8). Bypassing the need for IspG and IspH, by engineering the organisms to utilize the eukaryotic Fe-S protein-independent mevalonate pathway for isoprenoid synthesis, circumvents the necessity for Fe-S biosynthesis for survival (9).

## BUILDING IRON-SULFUR CLUSTERS

Because of the toxic nature of free Fe^2+^ and sulfide (S^2−^), tightly controlled mechanisms have evolved to synthesize Fe-S clusters from their monoatomic precursors, thereby minimizing the cytosolic concentrations of these elements not ligated to macromolecules (10). Three multiprotein assembly systems (nitrogen fixation [NIF], sulfur mobilization [SUF], and iron-sulfur cluster [ISC]) have been described in bacteria and archaea for the synthesis of Fe-S clusters for the assembly of [2Fe-2S] and [4Fe-4S] proteins (11–13). These systems function similarly, but they are biochemically discrete. Additional systems have been described for building more complex Fe-S clusters such as those found in dinitrogenase reductases and hydrogenases (reviewed in references 14 and 15).

The NIF system was the first described Fe-S synthesis system. NIF was discovered because it is essential for nitrogen fixation (13). NIF functions to provide basic Fe-S clusters for nitrogenase maturation, and it is often found in diazotrophs (16). SUF and ISC are responsible for building the Fe-S clusters for the maturation of the majority of nonnitrogenase Fe-S proteins. Bioinformatic analyses have identified the SUF system as the most prevalent machinery in prokaryotic genomes (17). Bacterial genomes can encode one (Staphylococcus aureus), two (Escherichia coli), or all three (Erwinia chrysanthemi and some nitrogen-fixing cyanobacteria) of the synthesis systems (17, 18) (see the section on regulation, below).

**FIG 1:**
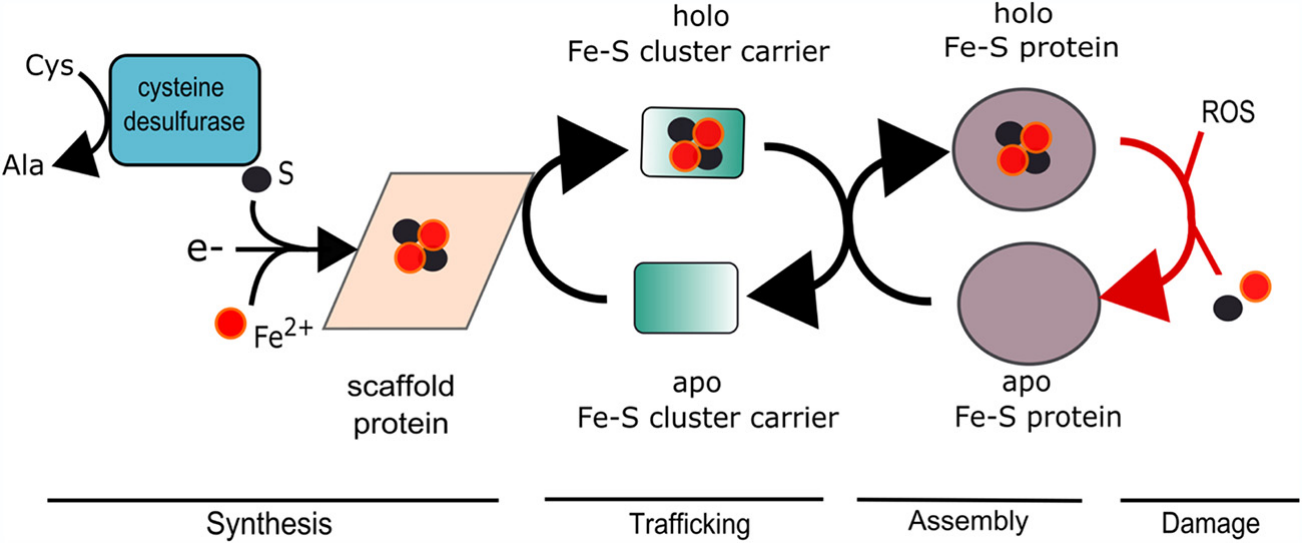
General mechanism of bacterial Fe-S protein assembly. Monoatomic Fe^2+^ and S^0^ are combined with electrons on a proteinaceous molecular scaffold forming an Fe-S cluster. The Fe-S cluster is transferred to one or more carrier proteins before being transferred to an apo-protein forming a holo-protein. Reactive oxygen species (ROS) can either damage the Fe-S cluster, which can subsequently be repaired, or destroy it, resulting in apo-protein formation.

**FIG 2:**
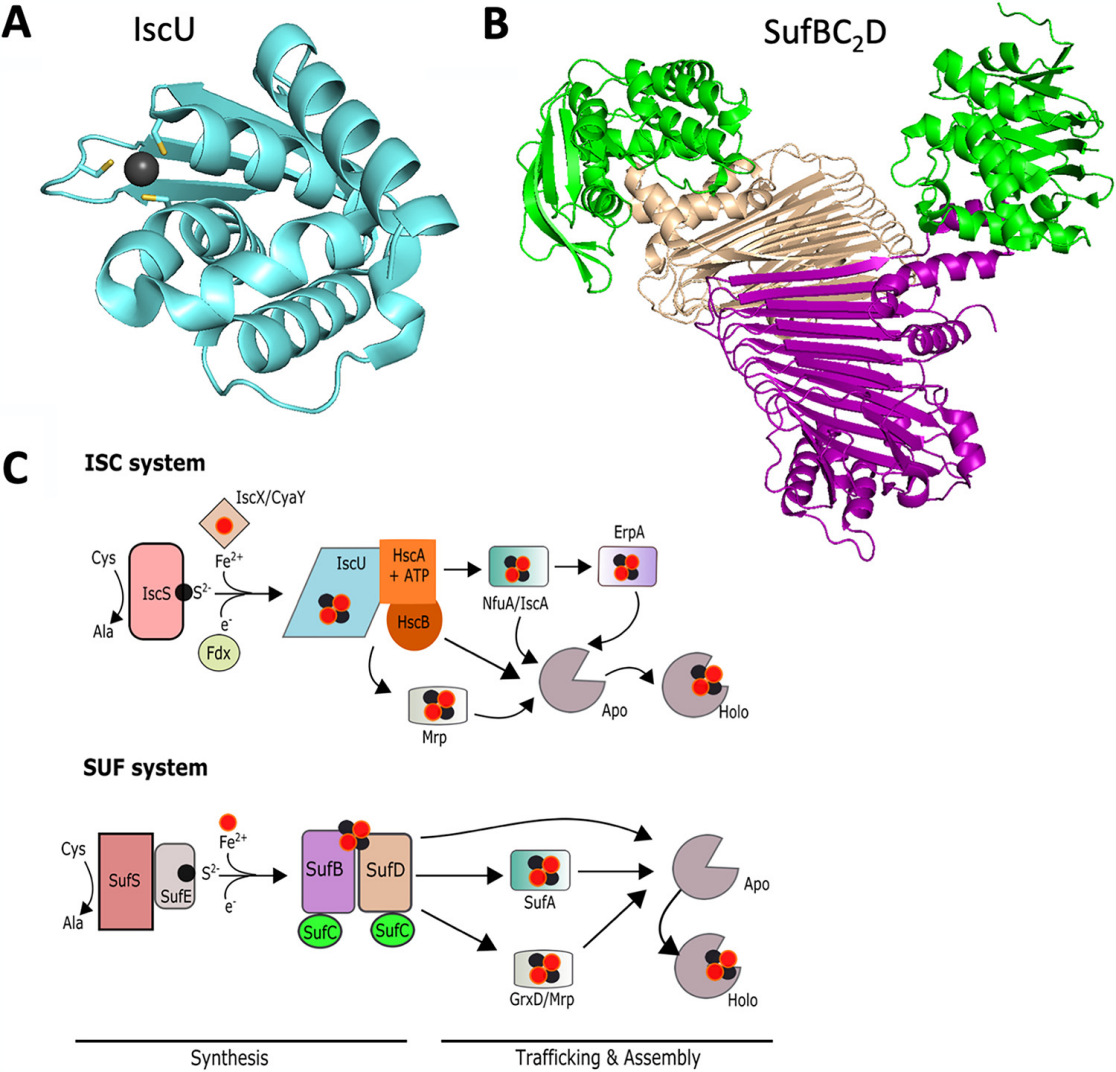
Iron-sulfur cluster synthesis. (A) Structure of IscU from Thermus thermophilus (PDB accession number 2QQ4). The gray ball is a Zn(II) ion, and the side chains of the three ligating cysteines are highlighted. (B) Structure of SufBC_2_D from Escherichia coli (PDB accession number 5AWF). SufC is shown in green, and SufB and SufD are shown in purple and tan, respectively. (C) Working models for ISC- and SUF-directed iron-sulfur protein maturation in Escherichia coli.

The SUF, NIF, and ISC macromolecular machines all use a common strategy to synthesize Fe-S clusters (Fig. 1). Iron, sulfur, and electrons are combined upon a cytosolic molecular scaffolding protein(s) to form an Fe-S cluster. SufBCD, IscU, and NifU are the scaffold proteins for the SUF, ISC, and NIF systems, respectively (Fig. 2) (16, 19, 20). Although the Suf proteins can be isolated with various ratios, it is thought that the active form of the SUF system has the ratio of one SufB, two SufC, and one SufD (SufBC_2_D) (Fig. 2B) (21, 22). The SufBD heterodimer interface may be the site of Fe-S cluster synthesis (22).

### Sulfur.

Sulfur is typically mobilized from a free cysteine (Cys) by pyridoxal phosphate (PLP)-dependent cysteine desulfurases (SufS, IscS, and NifS) (13). Cysteine desulfurases form a covalent persulfide intermediate and alanine as a by-product (23). The persulfide can subsequently be transferred to the synthesis machinery scaffold directly or through a surrogate carrier molecule (i.e., SufU or SufE) (19, 24). Thus far, the only described persulfide sulfur carrier molecules are associated with SUF systems. Biochemical analyses suggest that these persulfide carrier proteins allow controlled delivery that protects the system from poisoning by oxidants, such as hydrogen peroxide, which would be deleterious to sulfur transfer (25, 26). SufU and SufE act as persulfide carriers for SufBC_2_D in Bacillus subtilis and E. coli, respectively. SufU was initially thought to be a scaffold protein because of its ability to bind Fe-S clusters and its homology to IscU and NifU; however, biochemical analyses of the SufS-SufU complex demonstrated a unique sulfur transfer mechanism dependent on a zinc ligand from SufU (24, 27). Although the SufU and SufE primary amino acid sequences differ, they can individually act as protective persulfide carriers for SufBC_2_D; however, a *suf* operon usually codes for only one, suggesting that this functionality may have evolved twice. Some archaea such as Methanococcus maripaludis lack homologs of cysteine desulfurases. When this archaeon was cultured with ^35^S^2−^, there was an enrichment of ^35^S^2−^ in Fe-S cluster-containing proteins but not in free Cys, suggesting that sulfide, and not Cys, is the source of the sulfur for Fe-S synthesis (28).

### Electrons.

The *isc* operon typically encodes a [2Fe-2S] ferredoxin (Fdx), which can provide electrons for ISC-directed Fe-S synthesis. Fdx interacts with IscS, and reduced Fdx provides an electron to the IscS complex for sulfane (S^0^) reduction (29, 30). A second reduction event is required to produce S^2−^, which is the substrate for Fe-S cluster synthesis on IscU. NADH and NADP^+^-ferredoxin reductase can provide electrons for Fdx reduction *in vitro* (31). The electron donors to the scaffold proteins NifU and SufBC_2_D are unknown. SufBC_2_D copurifies with reduced flavin adenine dinucleotide (FADH_2_), consistent with the complex conducting redox chemistry (21). NifU, from the NIF system, contains a stable redox-active [2Fe-2S] cluster that may provide electrons for NIF-directed synthesis (32). The membrane-associated Rnf complex has a role in dinitrogen fixation in Azotobacter vinelandii, by donating electrons from NADH to ferredoxin using reverse electron flow and ΔμNa^+^ or ΔμH^+^ (33). Azotobacter vinelandii *rnf* mutants have a decreased capacity for dinitrogen reduction because of poor Fe-S cluster occupancy of the dinitrogenase reductase NifH (34). The *rnf* mutants also have decreased activity of the Fe-S enzyme aconitase. It is tempting to speculate that Rnf has a role in providing electrons for Fe-S cluster synthesis or repair.

### Iron.

The source of Fe for cluster building remains unknown. Several candidates such as CyaY and IscX have been proposed based on *in vitro* considerations, but subsequent *in vivo* investigations failed to provide supporting evidence. CyaY is the counterpart of mitochondrial frataxin. The reason why frataxin/CyaY was predicted to act as an iron donor came from (i) observing iron homeostasis disturbance in mitochondria from frataxin-deficient tissues or organisms and (ii) iron binding to CyaY *in vitro* although with weak affinity. Frataxin, in both eukaryotes and prokaryotes, forms a tripartite complex with the cysteine desulfurase NFS1/IscS and the scaffold ISU/IscU. *In vitro*, frataxin appears to have the opposite effect on Fe-S formation whether one studies the prokaryote (i.e., inhibition) or the eukaryote (i.e., stimulation) system (35). Possible explanations lie in differences in IscS (prokaryote) and NFS1 (eukaryote) cysteine desulfurase intrinsic biochemical features. In any case, studies *in vivo* in E. coli confirmed that CyaY is a positive effector of ISC-mediated Fe-S cluster biogenesis (36, 37).

The E. coli *isc* operon codes for IscX, which also binds Fe^2+^ with low affinity and has a role in ISC-directed Fe-S synthesis (36, 38). IscX binds to IscS at a location that overlaps the CyaY binding site (39). The presence of Fe^2+^ increases the affinity between IscX and IscU and stabilizes the complex (38). IscX associates with IscS-IscU, forming a tripartite complex resulting in inhibition of cysteine desulfurase activity. Analyses using both CyaY and IscX, in conjunction with the IscU-IscS complex, found that CyaY inhibits Fe-S cluster formation on IscU, which is mitigated by the addition of IscX at low Fe concentrations (<20 μM); however, the effect of IscX is negligible at higher concentrations (40).

While evidence suggests that IscA is an Fe-S cluster carrier (discussed below), one group of researchers found that E. coli IscA copurified with Fe but not sulfide (41, 151). Apo-IscA could be loaded with Fe^2+^ *in vitro* (*K_a_* = 3.0 × 10^−19^ M^−1^), and it bound one Fe per IscA. The Fe-loaded IscA could provide Fe for IscS-directed Fe-S cluster synthesis on IscU. An IscA_Y40F_ variant was defective in Fe^2+^ binding but appeared to bind an Fe-S cluster. *K_a_* values for Fe association, Fe-S cluster stability, Fe-S cluster transfer kinetics, and labile Fe and sulfide concentrations were not reported for the reconstituted IscA_Y40F_ variant (42). A wild-type *iscA* allele, but not an *iscA*_Y40F_ allele, could complement an *iscA* mutant. Whether IscA is a bona fide Fe donor *in vivo* remains to be established.

### Energy.

The assembly of Fe-S proteins can require an input of energy. SufC, of the SUF system, has both Walker A and Walker B nucleotide binding motifs and functions as an ATPase (43, 44). ATPase activity is stimulated by interaction with either SufD or SufB (45). The presence of SufC is necessary for SufB to interact with the sulfur transfer protein SufE (19). A conserved lysine (Lys40) in the Walker A motif is required for ATPase activity (46). The SufC_K40R_ variant interacts with SufB and SufD *in vitro*, but the *sufC*_K40R_ allele cannot replace *sufC*, suggesting that ATPase activity is necessary for SUF function (22). Consistent with these findings, the SufBC_2(K40R)_D variant does not assemble an Fe-S cluster *in vivo*, whereas SufBC_2_D does (47).

In the ISC system, the scaffold IscU interacts with an Hsp70-like chaperone (HscA) and a J-protein cochaperone (HscB) (48). Hsp70 chaperones have an ATP binding domain and a protein substrate binding domain. Biochemical and biophysical studies found that interactions between IscU and HscAB aid in building the Fe-S cluster on IscU and/or the transfer of the Fe-S cluster from holo-IscU to a target apo-protein (49). The proposed role of the cochaperone HscB is to escort IscU to HscA-ATP and promote ATP hydrolysis (50). After ATP hydrolysis, HscB is released because it has a low affinity for HscA-ADP, and IscU is prompted to deliver the Fe-S cluster to an apo-protein or Fe-S cluster carrier protein (49). ADP release by HscA induces conformational changes that promote IscU release, prompting a new cycle of chaperone-mediated Fe-S cluster synthesis and transfer (48, 51). The roles of HscAB were recently reviewed (52).

## DELIVERY OF IRON-SULFUR CLUSTERS TO TARGET APO-PROTEINS

After Fe-S cluster construction on a scaffolding protein, it is passed to a client apo-protein, forming the holo-protein. *In vitro* evidence for direct Fe-S cluster transfer from a scaffold to an apo-protein was demonstrated, but *in vivo* observations stress the essential role of carriers and cast doubt on the physiological relevance of the direct scaffold–apo-protein connection (41, 53–55).

Fe-S cluster carriers have been shown to bind [4Fe-4S] clusters, [2Fe-2S] clusters, or both (56). It is thought that the carriers typically deliver the Fe-S cluster that they are provided; however, the Fe-S carrier IscA (A-type carrier [ATC]) from Azotobacter vinelandii can bind both [4Fe-4S]^2+^ and [2Fe-2S]^2+^ clusters and can convert the [2Fe-2S]^2+^ form to the [4Fe-4S]^2+^ form using two-electron reductive coupling (56). It was proposed that the [4Fe-4S]^2+^ form could cycle back to the [2Fe-2S]^2+^ form by dioxygen-catalyzed cleavage, but this has not been experimentally demonstrated. Whether these cluster dynamics have a physiological role is unknown.

The number of carriers varies within different bacterial species. For instance, E. coli synthesizes at least six carriers (the A-type carriers [IscA, SufA, and ErpA], NfuA, GrxD, and Mrp), while Bacillus subtilis and Staphylococcus aureus have only two characterized carriers (SufA and Nfu). Strains lacking one or more Fe-S cluster carriers can often maintain the assembly of Fe-S proteins, suggesting that, in general, the carriers have a degree of functional overlap (53, 57). Note, however, that *erpA* is essential for aerobic growth in E. coli (9).

The number of targets exceeds the number of carriers, raising questions about the dynamics and specificity of the delivery network. While *in vitro* transfer assays have provided evidence that carriers can transfer clusters to apo-proteins, they did not reveal substantial insights into substrate specificity (41). In E. coli, the six carriers are not synthesized to the same levels, and expression varies based upon growth state and condition, suggesting that genetic control is key for orchestrating functional redundancy (58). We briefly discuss some of these intermediate actors below.

### A-type carriers.

**FIG 3:**
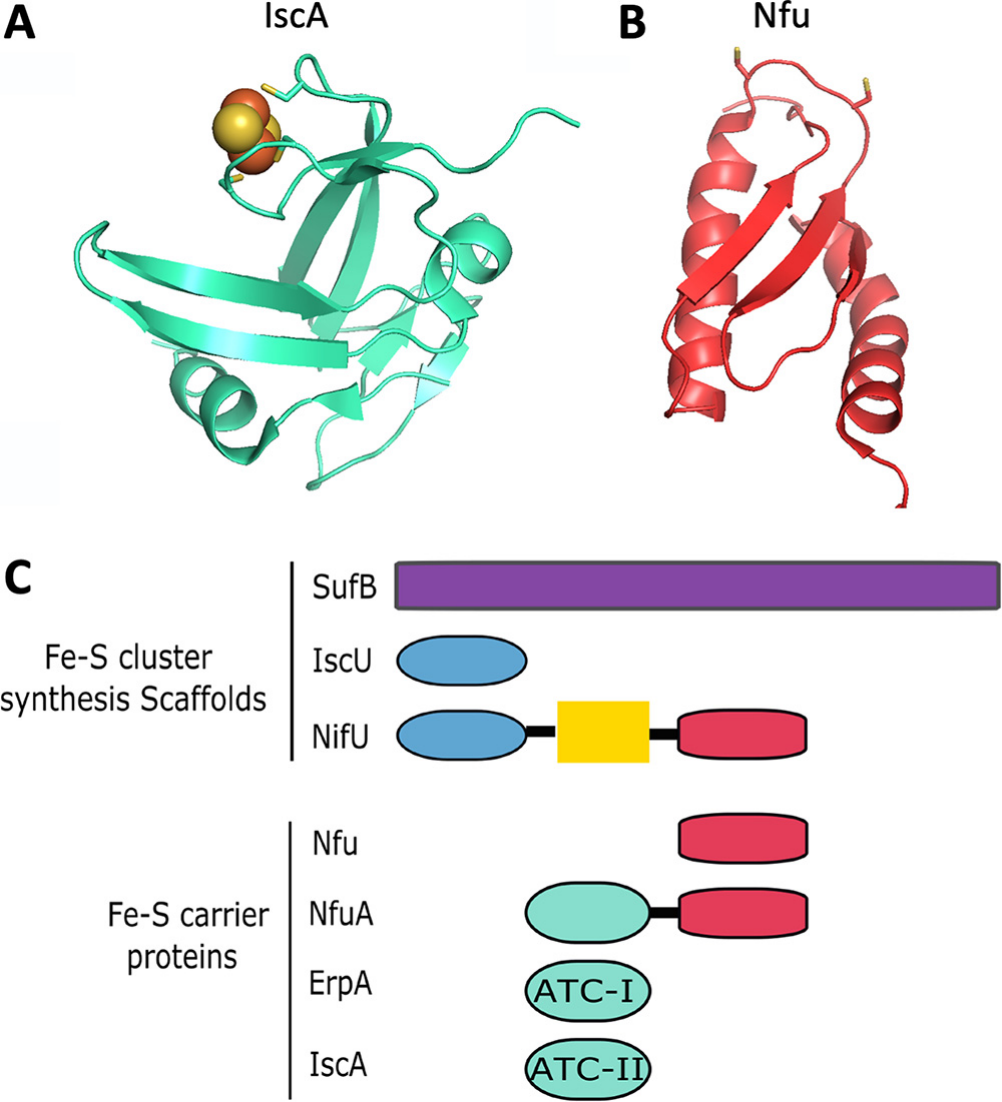
Iron-sulfur cluster carriage. (A) Structure of the A-type carrier IscA from Thermosynechococcus elongatus (PDB accession number 1X0G) with a [2Fe-2S] cluster bound. (B) Structure of Nfu from Staphylococcus epidermidis (PDB accession number 1XHJ). The cysteine thiols that are proposed iron-sulfur cluster ligands are highlighted. (C) Schematic representation of iron-sulfur cluster scaffolds and carriers.

A-type carriers (ATCs) are predicted to bind Fe-S clusters using three cysteine ligands. An X-ray structure of Thermosynechococcus elongatus IscA ligating a [2Fe-2S] cluster is illustrated in Fig. 3A (59). The three cysteinyl ligands (C37, C101, and C103) to the Fe-S cluster are highlighted. Phylogenetic analysis allowed researchers to divide the ATCs into two groups: ATC-I and ATC-II (53). Further functional studies showed that ATC-I proteins directly interact with the apo-proteins, while the ATC-II proteins associate with the scaffold (53). In E. coli, ErpA belongs to the ATC-I family, and IscA and SufA are of the ATC-II family. ErpA interacts physically with apo-proteins, whereas SufA and IscA, to an appreciable degree, do not. This suggests that the ATC-II carriers pass their Fe-S clusters to ATC-I for delivery to their final destinations (60). However, this simple view, derived mostly from normal growth conditions, might change under stress conditions. Indeed, SufA is predicted to interact directly with targets under such conditions, while ErpA could also interact with another type of carrier, NfuA, to form a stress-resistant heteromer (60). Fe-S clusters on holo-ErpA, holo-IscA, and holo-SufA are equally stable in the presence of dioxygen; however, the addition of the Fe-S cluster carrier NfuA to holo-ErpA nearly doubled the half-life of the ErpA Fe-S cluster (60). These data suggest that NfuA and ErpA may work in conjunction to form an oxidant-resistant Fe-S cluster delivery system. Altogether, the associations between NfuA and ErpA, as well as ErpA and apo-target proteins, support the hypothesis that under balanced aerobic conditions, ErpA conducts the last step of Fe-S cluster delivery in E. coli. As a matter of fact, with the exception of SoxR, the *in vivo* maturation of all target proteins studied depended upon ErpA (see below) (61).

### Nfu-type carriers.

The C-terminal domain of the A. vinelandii NifU Fe-S cluster scaffold is referred to as Nfu (Fig. 3B). The Nfu domain was demonstrated to bind and transfer a [4Fe-4S]^2+^ cluster to an apo-protein (62). An alternate protein, named NfuA, contains a C-terminal domain with homology to the Nfu domain of NifU and an N-terminal domain with homology to an A-type carrier (Fig. 3C). The NfuA A-type domain is referred to as “degenerated” because it lacks a cysteine required for Fe-S cluster ligation (63, 64). The C-terminal Nfu domain, but not the degenerated A-type domain, binds a [4Fe-4S]^2+^ cluster and can transfer this cluster to apo-proteins. The Fe-S cluster bound by holo-NfuA is more stable in the presence of dioxygen than that of holo-A-type carriers, suggesting a role for NfuA under oxidative stress conditions (60). Consistent with this hypothesis, strains lacking *nfuA* are defective in maturating Fe-S proteins facing oxidative stress (63, 64). Both the Nfu and A-type domains are necessary for NfuA function *in vivo* (63, 64). The A-type domain was demonstrated to function in targeting NfuA to apo-proteins (65, 66).

Three additional bacterial proteins consisting solely of an Nfu domain have been described. Nfu was necessary for the proper maturation of the Fe-S protein photosystem complex I (PS1) in *Synechococcus* and the maturation of several Fe-S proteins in Staphylococcus aureus and Helicobacter pylori (57, 67, 68). Holo-Nfu is a dimer with a bridging [2Fe-2S]^2+^ or [4Fe-4S]^2+^ cluster. Holo-Nfu could activate apo-PS1 and apo-aconitase. An S. aureus *nfu* mutant had decreased virulence in murine models of infection and decreased survival in neutrophils. An H. pylori *nfu* mutant was defective in colonizing murine stomachs.

### Mrp-type carriers.

Genetic studies identified *apbC*, encoding a member of the Mrp class, as being necessary to maturate the Fe-S enzymes ThiH and/or ThiC in Salmonella enterica (69). Biochemical studies demonstrated that ApbC can bind and effectively transfer Fe-S clusters to apo-proteins (70). ApbC is a dimer, and each monomer contains 2 cysteines separated by 2 amino acids (C-X-X-C motif). These cysteines are thought to provide four ligands, two from each monomer, for the ligation of a [4Fe-4S] cluster that bridges the dimer interface. Mrp proteins contain Walker A and B ATP hydrolysis motifs. An ApbC_K116A_ variant was defective in ATP hydrolysis and inactive *in vivo*. The addition of ATP did not accelerate ApbC-directed cluster transfer *in vitro*, and the ApbC_K116A_ variant proficiently transferred Fe-S clusters. These data led to the hypothesis that ATP hydrolysis is required for loading ApbC with an Fe-S cluster (71). ApbC was required for growth on the carbon source tricarballylate presumably because it functions in assembling the Fe-S enzyme tricarballylate reductase (TcuB) (72). The absence of ApbC could be bypassed by increasing the expression of *iscU* or by decreasing tricarballylate influx and thereby preventing tricarballylate accumulation, which inhibits isocitrate dehydrogenase (73, 74).

### Monothiol glutaredoxins.

As the name suggests, monothiol glutaredoxins lack the traditional dithiol C-X-X-C motif and instead have a C-G-F-S motif. E. coli *grxD* encodes a monothiol glutaredoxin (75). Combining a *grxD* mutation with an *iscU* mutation resulted in synthetic lethality, suggesting that GrxD functions in conjunction with the SUF machinery to assembly Fe-S proteins (E. coli must have functional SUF or ISC for viability [7]). GrxD purified from E. coli contained a [2Fe-2S] cluster. To chemically reconstitute an Fe-S cluster on apo-GrxD, the reaction mixture required glutathione (GSH) (76). GrxD binds a [2Fe-2S] cluster that bridges a homodimer interface using one cysteine ligand from each monomer, and GSH thiolates provide two additional ligands (77). The holo-GrxD homodimer can transfer an Fe-S cluster to apo-Fdx, forming the [2Fe-2S] holo-Fdx. GrxD was recently shown to cooperate with NfuA in the maturation of the Fe-S enzyme MiaB (78).

E. coli BolA is an ortholog of Saccharomyces cerevisiae Fra2, which forms a heterodimer with a monothiol glutaredoxin to bind a [2Fe-2S] cluster (79, 80). When purified from E. coli, BolA copurifies with GrxD and vice versa. An Fe-S cluster could not be reconstituted on BolA; however, an Fe-S cluster could be reconstituted on the BolA-GrxD heterodimer, and the cluster could be transferred to apo-Fdx (76). E. coli *bolA* and *grxD* mutants do not phenocopy one another, suggesting that they can also function independently.

## AUXILIARY FACTORS UTILIZED IN IRON-SULFUR PROTEIN MATURATION

Several loci have been identified that function in the assembly of Fe-S proteins but are not considered part of the core Fe-S cluster biosynthetic apparatus. These factors are not typically found within operons encoding the core ISC, SUF, or NIF machineries.

### SufT.

The *sufT* gene is often associated with *suf* operons (defined by having *sufB* and *sufC*) in bacterial and archaeal genomes (81). Typically, SufT proteins, such as those encoded by S. aureus and B. subtilis, are composed entirely of domain of unknown function 59 (DUF59). Larger proteins containing a DUF59 domain have roles in Fe-S cluster assembly, including the eukaryotic cytosolic Fe-S cluster assembly (CIA) factor CIA2, which functions in the maturation of nuclear and cytosolic Fe-S proteins (82–84). S. aureus strains lacking SufT have decreased activities of Fe-S enzymes under conditions requiring a high demand for Fe-S clusters. The phenotypes associated with the Δ*sufT* and Δ*nfu* mutations were synergistic (81, 85). Moreover, the overproduction of Nfu mitigated the phenotypes of the Δ*sufT* strain. These data suggest that SufT functions in Fe-S carriage and has some degree of functional overlap with Nfu; however, Fe-S cluster binding by SufT remains elusive. SufT was reported to be essential in Mycobacterium tuberculosis (86).

### Low-molecular-weight thiols.

The role of low-molecular-weight (LMW) thiols in Fe-S protein assembly is likely multifaceted and they could function in all four steps of Fe-S protein assembly: biogenesis, trafficking, assembly, and repair. In eukaryotes, GSH has been associated with the synthesis and trafficking of Fe-S clusters from the mitochondrion to the cytosol (71, 87). In bacteria, genetic and biochemical studies demonstrated a role for GSH in assembling Fe-S proteins, in addition to its roles in maintaining proper intracellular redox (88). GSH can act as an Fe buffer by binding nonincorporated cytosolic Fe (79, 89). GSH can also provide electrons to reduce Fe^3+^ to Fe^2+^ (90). GSH, in conjunction with monothiol glutaredoxins, delivers Fe-S clusters to the apo-protein targets (77, 80, 91). GSH can bind and deliver Fe-S clusters *in vitro*, but the *in vivo* relevance of this chemistry is unknown (92). GSH can also reduce oxidized protein cysteine residues before Fe-S cluster insertion.

Many microorganisms, including S. aureus, do not produce GSH but instead produce the LMW thiol bacillithiol (BSH) (93). An S. aureus strain defective in producing BSH exhibits phenotypes similar to those of cells lacking Fe-S cluster carriers, including decreased activities of Fe-S-dependent enzymes (94). The phenotypes of a BSH-minus strain were suppressed by the multicopy expression of *sufA* or *nfu* but not by the overexpression of the SUF system. These data suggest that the phenotypes of a BSH-minus strain were not the result of faulty *de novo* Fe-S synthesis but rather were the result of defective assembly or repair of Fe-S proteins (95). A strain lacking BSH did not appear to suffer from decreased reactive oxygen species (ROS) metabolism, but a protective role for BSH in buffering against metal ion poisoning of Fe-S enzymes or the maturation machinery has not been ruled out (96).

### Folic acid binding protein (YgfZ).

E. coli YgfZ (COG0354) is a homolog of yeast Iba57p, which is a mitochondrial protein that participates in the assembly of mitochondrial Fe-S proteins (97). A Δ*ygfZ* mutant strain has decreased activities of selected Fe-S enzymes, including MiaB, and is sensitive to ROS stress (98). YgfZ binds tetrahydrofolate (THF), and an E. coli strain lacking the ability to synthesize folate has MiaB activity similar to that of a Δ*ygfZ* mutant, suggesting that folate, as well as YgfZ, is utilized in assembling some Fe-S proteins. The COG0354 proteins are paralogous to enzymes that utilize THF to accept formaldehyde units, leading to the hypothesis that YgfZ functions to remove one-carbon units that deleteriously affect the functions of Fe-S proteins (99).

### RIC proteins.

E. coli *ytfE* encodes a repair of iron clusters (RIC) protein that has increased expression during nitric oxide stress (100). The expression of *ytfE* is directly controlled by NsrR, which directly responds to nitric oxide (NO^·^) levels (101, 102). An E. coli *ytfE* mutant is sensitive to NO^·^ or H_2_O_2_ stress, and Fe-S enzymes have decreased activities after cell extracts from the *ytfE* mutant are treated with hydrogen peroxide (H_2_O_2_) or NO^·^. Importantly, damaged Fe-S proteins had a lower rate of repair in the *ytfE* mutant (103). YtfE and its homologs are di-Fe hemerythrin-like proteins (104, 105). The Fe atoms of holo-YtfE are labile and can be used as an Fe source for Fe-S cluster synthesis *in vitro* (106). These findings resulted in renaming these proteins “repair of iron clusters” (RIC). The mechanism by which RIC proteins may repair damaged Fe-S clusters is unknown. YtfE interacts with the Fe scavenger Dps *in vivo*, and their corresponding genes have genetic interactions. These findings led to the hypothesis that Dps may be providing Fe to YtfE to be used for the repair of damaged Fe-S clusters (107). In S. aureus, *ytfE* (*scdA*) and *dps* protect against H_2_O_2_ damage, and both are transcriptionally regulated by SrrAB, which responds to electron flux through respiratory pathways (108, 109).

Physiological, genetic, and biochemical data suggest that a *ytfE* mutant has more NO^·^-induced damage and reduced activity of the Fe-S cluster-utilizing transcription factor (TF) NsrA. Structural data show that YtfE has a hydrophobic channel where NO^·^ could access the Fe ions, and the Fe atoms have been shown to ligate NO^·^ (105, 110). These data support the hypothesis that YtfE functions in *S*-*trans*-nitrosylation or the removal of NO^·^ from nitrosylated proteins (111). However, YtfE was not able to release NO^·^ from nitrosylated fumarase, a [4Fe-4S]-requiring dehydratase. YtfE contributes to Yersinia pseudotuberculosis and Haemophilus influenzae pathogenesis (112, 113).

## REGULATION OF IRON-SULFUR CLUSTER SYNTHESIS

Fe-S cluster biosynthesis is controlled by regulators that sense environmental conditions potentially adverse for Fe-S cluster assembly, such as Fe limitation or oxidative and nitrosative stress, which affect the stability and integrity of the cofactors. Integrating these stimuli with Fe-S synthesis ensures that demand is met and fitness is maintained.

### The situation in E. coli: adapting to fluctuating conditions and switching between machineries.

In E. coli, Fe-S cluster biosynthesis is achieved by using two types of machineries, ISC and SUF, which permit maturation of the same set of apo-proteins under a wide breadth of growth conditions. Particularly, genetic control circuits occur that endow E. coli with the capacity to synthesize one or the other machinery under different growth conditions and thereby have the Fe-S cluster biogenesis capacity to match the Fe-S demand, regardless of the growth conditions. Two transcriptional regulators, IscR and Fur, and a noncoding RNA, RyhB, are key actors in orchestrating this adaptative response, as all three control, directly or indirectly, the expression of the *isc* and *suf* operons.

### (i) IscR-mediated Fe-S cluster homeostasis control.

**FIG 4:**
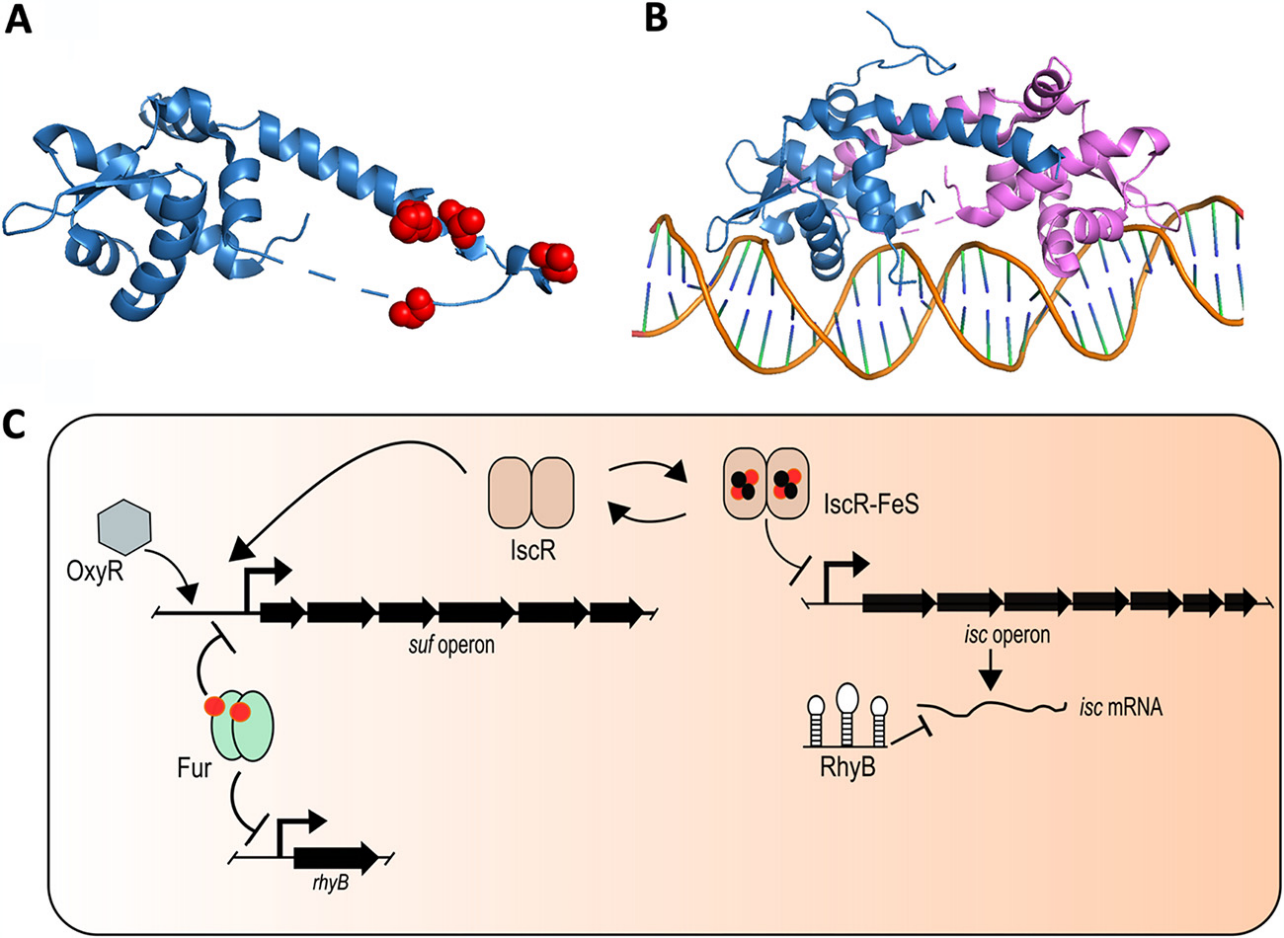
Regulation of iron-sulfur cluster synthesis in Escherichia coli. (A) X-ray structure of an apo-IscR monomer with the proposed Fe-S cluster ligands (C92A, C98A, C104A, and H107) highlighted in red (PDB accession number 4HF1). Note that in this IscR variant, the ligating cysteines have been changed to alanines. (B) X-ray structure of dimeric apo-IscR bound to the *hya* promoter, which is a type 2 binding site (PDB accession number 4HF1). Each monomer is differently colored (blue and pink). (C) Model for the regulation of ISC and SUF expression in Escherichia coli.

IscR is a TF that belongs to the Rrf2 family of winged helix-turn-helix TFs. It hosts a [2Fe-2S] cluster, which allows sensing aerobiosis, oxidative stress, iron limitation, and, possibly, reactive nitrogen species (RNS). Mutagenesis and structural studies have identified residues Cys92, Cys98, Cys104, and His107 as Fe-S cluster ligands (Fig. 4A) (114, 115). A His ligand is uncommon, and this might render the cluster labile and sensitive to stress signals. It is particularly useful for the IscR regulator since its activity is not influenced by the oxidative state of its cluster but is strictly dependent on the presence/absence of the cluster (115, 116). IscR is found in the apo- and holo-forms, and both types can have regulatory functions (117).

Two types of binding sites, types 1 and 2, are found within IscR-regulated promoters. Holo-IscR binds type 1 sites, and the holo- and apo-forms bind the type 2 sites (Fig. 4B) (114). The type 1 inverted repeat sequence is well conserved and is mainly found upstream of genes encoding Fe-S building proteins (the *isc*, *erpA*, and *nfuA* loci) (118). The type 2 sequence is an imperfect palindrome and highly degenerated, which causes large variations in binding affinities between operator regions (118). Interestingly, while the type 1 sequence-containing promoters are all repressed by IscR, genes preceded by a type 2 sequence can be either repressed or activated by IscR. This does not correlate with the position of the IscR binding site, as shown with the *hyaA* and *sufA* genes. Both promoters exhibit a type 2 sequence within their −35 consensus promoter sequence yet show opposite expression patterns as *hyaA* expression is repressed by holo-IscR, whereas *sufA* expression is activated by apo-IscR (118).

In E. coli, *iscR* is the proximal gene in the *isc* operon and is separated from the next gene (*iscS*) by an unusually long untranslated region that is targeted by RyhB, a noncoding RNA (see below). Under balanced conditions, IscR is maturated by the ISC machinery, and holo-IscR acts as a repressor of its own expression as well as the expression of downstream *isc* genes until equilibrium shifts toward a low level of apo-IscR. At this point, repression is alleviated, more IscR is synthesized, and the ISC machinery is produced, resulting in an increased level of holo-IscR in the cell. A feedback loop is then set up, and holo-IscR represses its own expression as well as those of the following *isc* genes. Upon an increase in the cellular demand for Fe-S cluster synthesis (iron limitation and oxidative stress), there is competition between apo-protein substrates and newly synthesized apo-IscR for the ISC machinery. Next, apo-IscR accumulates, and this form activates the expression of the *suf* operon (119). In summation, IscR represses the transcription of *isc* and activates *suf* transcription in its Fe-S-bound and unbound forms, respectively, directly connecting both the cell’s Fe-S cluster biogenesis capacity and Fe-S cluster demand (Fig. 4C).

Evidence has been provided that the efficiency of ISC proteins, in particular IscU, would be lowered under stress conditions, opening the possibility that the contribution of the ISC machinery declines under such conditions and that the cell would rather switch from ISC to SUF rather than accumulating both (25). Importantly, IscR appears to be a poor substrate for the SUF system, and therefore, IscR is likely to remain mostly in its apo-form if the cell thrives under stress conditions (54). The iron-responding Fur-RhyB genetic circuit also favors such a switch (see below).

### (ii) Iron-mediated control of Fe-S biogenesis by Fur and RyhB.

Iron availability is sensed by the transcriptional regulator Fur, which represses the synthesis of the noncoding RNA RyhB, among others. Because both Fur and RyhB regulate the expression of the *isc* and *suf* operons, directly or indirectly, they are likely to contribute to the switch between the ISC and SUF machineries as a mode of adaptation to iron bioavailability. Holo-Fur acts as a repressor of *suf* operon transcription. Under iron-limiting conditions, the Fe^2+^ cofactor of Fur is lost, and repression is alleviated, providing an opportunity for IscR-dependent *suf* operon expression (120). Meanwhile, *ryhB*, which is also repressed by holo-Fur, is expressed and targets the intergenic region between *iscR* and *iscS*, causing translation inhibition of the downstream *iscSUA* genes and probably mRNA decay, whereas a stem-loop structure forms, enabling the stabilization of the upstream *iscR* messenger moiety (Fig. 4C) (121). Under these conditions, apo-IscR accumulates and activates the expression of the *suf* operon. Consequently, iron limitation enhances the expression of the *suf* operon by both alleviating Fur repression and favoring apo-IscR activation, while the expression of *iscSUA* is shut off by RyhB-mediated translation inhibition and possibly the poor activity of the encoded IscU scaffold protein. It should be noted that a recent study suggests that Fur senses iron homeostasis by binding a [2Fe-2S] cluster instead of Fe^2+^ as is currently suggested (122). If this observation were to be confirmed *in vivo*, it would link the Fur-repressing activity to both iron availability and Fe-S biogenesis, in which case the interplay between IscR and Fur would be an important issue to decipher *in vivo*.

### (iii) Switching the Fe-S cluster synthesis machineries under redox stress.

The OxyR transcriptional regulator activates the expression of *suf* genes in response to H_2_O_2_ (Fig. 4C). The OxyR binding site is located far upstream from the *sufA* promoter (operon *sufABCDSE*), and OxyR-dependent activation requires the binding activity of IHF to bring the OxyR site closer to the −10 and −35 promoter elements (123). OxyR- and apo-IscR-mediated activations have been found to be additive (119). Thus, because oxidative stress could well favor the shift from holo-IscR to apo-IscR, it is possible that under such conditions, E. coli accumulates the synthesis of both ISC, following the alleviation of IscR repression, and SUF, following activation by OxyR. However, oxidative stress is known to lower iron bioavailability (by oxidizing Fe^2+^ to Fe^3+^). This could alleviate holo-Fur-dependent repressions, leading to *ryhB* expression and, subsequently, *isc* repression, thereby preventing the accumulation of both systems. Moreover, IscU activity was reported to be altered by oxidative stress (25). Thus, under oxidative stress, a genetic switch, like that observed under iron-limiting conditions, might prevail. The expression of the *suf* operon is also repressed by the [4Fe-4S] cluster-containing transcription factor NsrR under normal growth conditions. Under nitrosative stress conditions, the Fe-S cluster is lost, and NsrR-dependent repression is alleviated (124). Again, because IscR could shift from holo to apo under such redox stress conditions, a shift between the machineries could take place.

### (iv) Switching between machineries promotes antibiotic tolerance.

One phenotypic consequence of the stress-controlled switch between the ISC and SUF systems is enhanced resistance to aminoglycoside antibiotics (125). The uptake of aminoglycosides is dependent upon the proton motive force (PMF), and therefore, the bactericidal activity of these antibiotics is proportional to respiration efficiency. Mechanistic causes of the aminoglycoside tolerance resulting from iron limitation are (i) the down- and upregulation of ISC and SUF, respectively, by the IscR- and Fur/RyhB-dependent controls described above; (ii) inefficient maturation of respiratory complexes I and II by the SUF system, resulting in decreased respiratory efficiency; and (iii) Fur/RyhB-dependent downregulation of complex I and II synthesis (125, 126).

Another illustration of the link between the switching machinery and antibiotic tolerance is demonstrated in the case of fluoroquinolones. Exposing E. coli to phenazine methosulfate (PMS), a redox-cycling compound that causes oxidative stress and NAD(P)H exhaustion, yielded enhanced tolerance to norfloxacin, a DNA gyrase inhibitor (61). Under PMS exposure, E. coli switches to the SUF system, which can target Fe-S clusters to the transcriptional activator SoxR. The Fe-S cluster bound to SoxR becomes oxidized and allows SoxR to activate *soxS* transcription. SoxS then activates the expression of *acrAB*, encoding an efflux pump, which exports fluoroquinolones out of the cell.

### A feed-forward loop mediated by IscR and RyhB.

Acting at both transcription and translation initiation permits finely tuned gene expression. An example is given by the dual control afforded by IscR and RyhB on the expression of *erpA* in E. coli. As mentioned above, E. coli synthesizes multiple Fe-S carriers, and whether they have a degree of functional redundancy has been a matter of debate. The transcription of *erpA* is repressed by holo-IscR, while *erpA* mRNA translation is negatively regulated by RyhB (58). These data led to the hypothesis that ErpA is synthesized under neither Fe-replete conditions (repression by IscR) nor Fe limitation conditions (inhibition by RyhB). This double control allows ErpA synthesis within a window of intermediate Fe concentrations. The added value of this double control is that ErpA is synthesized under conditions in which neither of the two other A-type carriers, SufA and IscA, is fully synthesized. This control ensures the continuing presence of at least one carrier throughout fluctuating iron concentrations (58).

### IscR as a sensor of the anaerobic/aerobic switch.

The regulation of gene transcription often involves multiple transcriptional regulators, which might compete (or synergize) for closely located operator sites and modify the importance of each other’s influence. The influence of IscR on alternate regulators is well documented by the unexpected role of IscR in cell-to-cell variability during the shift from dioxygen respiration to trimethyl amine oxide (TMAO) respiration. The genes that encode TMAO reductase are under the transcriptional control of the TorT/TorS/TorR three-component regulatory system (127, 128). In the presence of dioxygen, *torT-torS* expression is repressed by IscR, and the level of TorT/S is so low that a stochastic effect prevails, leading to cell-to-cell variability in TMAO reductase synthesis. In contrast, under anoxic TMAO-respiring conditions, IscR titers decrease, and *torT-torS* expression is derepressed. The levels of TorT/S are now high enough to cancel any effect from stochasticity in gene expression. Therefore, IscR is determining in this “regulated stochasticity” by acting upstream in the cascade, controlling the level of TorT/S, and mediating the oxygen regulation of cell-to-cell variability (129).

### The role of IscR in pathogenic bacteria.

IscR is widely conserved and was studied in several bacteria, including the pathogens Erwinia chrysanthemi (18), Pseudomonas aeruginosa (130), Burkholderia mallei (131), Vibrio vulnificus (132), Salmonella enterica (133), and Yersinia pseudotuberculosis (134). Because Fe-S-based biology is central to cellular bioenergetics and metabolism, it is expected to be important for bacterial fitness and multiplication within its host. Moreover, both iron limitation and oxidative stress are conditions met by pathogens during host colonization, suggesting that IscR may be instrumental in coordinating adaption.

Less expected, however, was that IscR would directly control the synthesis of key virulence determinants as was reported in both S. enterica and Yersinia pseudotuberculosis. Both pathogens rely on type 3 secretion systems (T3SSs) utilized to inject effectors into the host cells, and IscR controls the synthesis of the T3SSs in both bacterial species. S. enterica synthesizes two T3SSs, referred to as Salmonella pathogenicity island 1 (SPI1) and SPI2. SPI1 is required for the passage of the bacterium across the epithelial border, while SPI2 is required to establish an S. enterica-containing vacuole in macrophages. The *spi1* locus includes *hilD*, encoding a major virulence regulator, which controls its own synthesis and that of effectors. A type 2 IscR binding site is present upstream of *hilD*, and IscR binding was proposed to interfere with HilD positive autoregulation, thereby lowering virulence (133). Consistently, an *iscU* mutant, which has a high level of apo-IscR, exhibited a reduced invasion capacity in epithelial cells and attenuated virulence in a murine model of infection. Conversely, an *iscR* mutant was hyperinvasive in HeLa cells (133). In Y. pseudotuberculosis, IscR binds a type 2 motif within the promoter of a gene encoding the transcription factor LcrF. The *lcrF* gene is located in the virulence plasmid pYV, which also encodes a T3SS. LcrF regulates the transcription of the T3SS-secreted effector genes and, thereby, virulence. IscR was essential for T3SS-dependent secretion, and an *iscR* mutant was deficient in colonization of Peyer’s patches, spleen, and liver in murine models (134). In V. vulnificus, IscR directly activates the expression of the *vvhBA* genes encoding a cytolysin in response to host-derived signals such as nitrosative stress and iron starvation (135). In E. coli, some fimbria genes are directly regulated by IscR, such as *cfaA* and *fimE* (136, 137). Finally, IscR has been shown to coordinate oxidative stress resistance during pathogenesis in Pseudomonas aeruginosa and Xanthomonas campestris (130, 138).

### Regulation of Fe-S biogenesis by SufR.

SufR, first described in *Cyanobacteria*, is another Fe-S biogenesis-dedicated transcriptional regulator (139). Interestingly, while *Cyanobacteria* have the two main Fe-S biogenesis machineries, ISC and SUF (and sometimes the NIF system dedicated to nitrogenase maturation), the IscR regulator regulates the transcription of the *isc* locus only. The expression of the *suf* locus is under the transcriptional control of its own regulator, SufR. SufR belongs to the DeoR family of helix-loop-helix regulators. Its DNA binding domain is located in the N-terminal portion of the protein, and it has a nonconventional Fe-S binding site in the C-terminal portion (C-X_12_-C-X_13_-C-X_14_-C) where a [4Fe-4S] cluster is coordinated (140). Holo-SufR is a repressor of the *suf* locus, thereby downregulating its own expression. It binds a perfect palindromic sequence (CAAC-N_6_-GTTG) that is highly conserved in the promoter regions of *suf* loci in *Cyanobacteria* (140). SufR regulatory activity is sensitive to redox stress, oxidative stress, and iron starvation (139, 140). It is interesting to note that the SUF system appears to be the most important in *Cyanobacteria*, and all the genes of the *suf* locus are essential. This could be why a dedicated regulator controls *suf* expression. Most of the Gram-positive bacteria possess only the SUF system; however, SufR seems to be underrepresented, with only two examples described in *Actinobacteria*: Mycobacterium tuberculosis and Streptomyces avermitilis (141, 142). How the *suf* locus is regulated in most of the Gram-positive bacteria lacking SufR is unknown.

Overall, IscR and, to a lesser extent, SufR appear to have primary functions as regulators of Fe-S biosynthesis. IscR is conserved among the bacterial species producing an ISC machinery and coordinates Fe-S biosynthesis with other cellular functions, including pathogenesis. In contrast, SufR was found only in *Cyanobacteria* and some *Actinobacteria* and is dedicated to regulating the *suf* locus. Both regulators coordinate Fe-S biogenesis with Fe-S bioavailability, and they are assisted in this task by stress-specific regulators such as Fur for iron availability, OxyR for oxidative stress, and NsrR for nitrosative stress.

## IRON-SULFUR PROTEIN ASSEMBLY AS AN ANTIPATHOGEN TARGET

The susceptibility of bacteria to host-distributed chemicals such as copper (Cu) ions, ROS, and RNS, which act, in part, to poison the Fe-S cluster-requiring proteins, implies that higher eukaryotes have evolved to prevent bacterial growth by targeting Fe-S protein assembly (5, 143, 144). High-density transposon screens or directed-mutagenesis studies suggest that the assembly of Fe-S proteins is essential for many human bacterial pathogens (6). Importantly, microbes synthesize Fe-S clusters using machineries that are functionally similar but biochemically distinct from the machineries used by higher eukaryotes. Bacteria defective in maturating Fe-S proteins have decreased virulence or fitness in models of infection (6, 57, 145). An inability to assemble Fe-S proteins affects numerous metabolic pathways, resulting in metabolic chaos. These facts imply that Fe-S protein assembly is a viable target for antimicrobial therapy.

As an example, a small molecule called ‘882 decreased the activity of aconitase *in vivo* but not *in vitro* (146). A “pulldown” assay using immobilized ‘882 as bait found that it associates with SufBCD. SufC associated with ‘882 with a dissociation constant (*K_d_*) for ‘882 of ∼3 μM. These data led to the hypothesis that ‘882 inhibited Fe-S protein assembly by inhibiting Suf-dependent Fe-S cluster synthesis. The Suf system has also been proposed to be a target for other nonbacterial pathogens, including Toxoplasma gondii and Plasmodium falciparum (147). For the latter, the molecule d-cycloserine, which can form a covalent adduct with PLP, can inhibit the cysteine desulfurase SufS, resulting in growth inhibition (148).

## FUTURE DIRECTIONS

New Fe-S cluster assembly factors are continually being discovered, lending support to the hypothesis that additional factors exist and that our current knowledge is incomplete. To move forward, we need to broaden our approaches by using newly available techniques and expand the organisms studied. Studies using E. coli and A. vinelandii have provided the bulk of the information about how bacteria assemble Fe-S proteins. These Gram-negative organisms are relatively unique in the fact that they have more than one biosynthetic system, which are, for the most part, functionally redundant. In contrast, very few studies have been conducted on Fe-S cluster assembly in Gram-positive bacteria, which typically encode only one Fe-S cluster biosynthesis system.

Several questions remain about Fe-S protein maturation and its regulation. The Fe and electron donors for Fe-S cluster synthesis and repair remain elusive. We also do not fully understand the mechanism by which Suf synthesizes Fe-S clusters or the functions of many factors utilized for maturating Fe-S proteins. We need to increase our understanding of how the Fe-S cluster assembly machinery is integrated with metabolic pathways that require Fe-S proteins. It is not well understood if Fe-S cluster carriers transfer Fe-S clusters to all apo-targets with the same efficacy or if there is an apo-protein hierarchy driven by carrier specificity. Understanding this integration will provide insights into metabolite balance and the consequences of decreasing metabolic flux through a pathway that requires an Fe-S protein since it is a costly process for the cells (149). This knowledge will be important not only for medicine and the development of specific antipathogen targets but also for scientists using organisms to conduct green chemistry. Inefficient Fe-S protein maturation, such as in organisms engineered to produce biofuels, fix dinitrogen, or generate secondary metabolites, could decrease the yields and the efficiency of desired processes, ultimately decreasing profits and productivity (150).
